# Designs and Characterization of Subunit Ebola GP Vaccine Candidates: Implications for Immunogenicity

**DOI:** 10.3389/fimmu.2020.586595

**Published:** 2020-11-04

**Authors:** Valentina Agnolon, Divor Kiseljak, Maria J. Wurm, Florian M. Wurm, Charlotte Foissard, Fabrice Gallais, Sarah Wehrle, César Muñoz-Fontela, Laurent Bellanger, Bruno Emanuel Correia, Giampietro Corradin, François Spertini

**Affiliations:** ^1^ Division of Immunology and Allergy, Centre Hospitalier Universitaire Vaudois (CHUV), Lausanne, Switzerland; ^2^ ExcellGene SA, Monthey, Switzerland; ^3^ Faculty of Life Sciences, École Polytechnique Fédérale De Lausanne (EPFL), Lausanne, Switzerland; ^4^ Université Paris Saclay, Commissariat à l’Energie Atomique et aux énergies alternatives (CEA), Institut national de recherche pour l’agriculture, l’alimentation et l’environnement (INRAE), Département Médicaments et Technologies pour la Santé (DMTS), SPI, Bagnols-sur-Cèze, France; ^5^ Laboratory of Protein Design and Immunoengineering, École Polytechnique Fédérale De Lausanne (EPFL), Lausanne, Switzerland; ^6^ Bernhard Nocht Institute for Tropical Medicine, Hamburg, Germany; ^7^ German Center for Infection Research (DZIF), Partner site Hamburg, Hamburg, Germany; ^8^ Department of Biochemistry, Université de Lausanne (UNIL), Epalinges, Switzerland

**Keywords:** Ebola virus, glycoprotein, Ebola glycoprotein, trimeric protein, vaccine, recombinant vaccine

## Abstract

The humoral responses of Ebola virus (EBOV) survivors mainly target the surface glycoprotein GP, and anti-GP neutralizing antibodies have been associated with protection against EBOV infection. In order to elicit protective neutralizing antibodies through vaccination a native-like conformation of the antigen is required. We therefore engineered and expressed in CHO cells several GP variants from EBOV (species *Zaire ebolavirus*, Mayinga variant), including a soluble GP ΔTM, a mucin-like domain-deleted GP ΔTM-ΔMUC, as well as two GP ΔTM-ΔMUC variants with C-terminal trimerization motifs in order to favor their native trimeric conformation. Inclusion of the trimerization motifs resulted in proteins mimicking GP metastable trimer and showing increased stability. The mucin-like domain appeared not to be critical for the retention of the native conformation of the GP protein, and its removal unmasked several neutralizing epitopes, especially in the trimers. The soluble GP variants inhibited mAbs neutralizing activity in a pseudotype transduction assay, further confirming the proteins’ structural integrity. Interestingly, the trimeric GPs, a native-like GP complex, showed stronger affinity for antibodies raised by natural infection in EBOV disease survivors rather than for antibodies raised in volunteers that received the ChAd3-EBOZ vaccine. These results support our hypothesis that neutralizing antibodies are preferentially induced when using a native-like conformation of the GP antigen. The soluble trimeric recombinant GP proteins we developed represent a novel and promising strategy to develop prophylactic vaccines against EBOV and other filoviruses.

## Introduction

The Ebola virus (EBOV) disease (EVD) epidemic occurring in Democratic Republic of Congo has been declared, with an overall case-fatality ratio of 66%, a Public Health Emergency of International Concern by the WHO in July 2019 (1). The recently licensed vaccine against Ebola is based on a live-attenuated recombinant rVSV expressing EBOV glycoprotein (GP), it is recognized to be safe and highly efficacious after a single-dose injection, and it is currently being adopted in the field in a ring-vaccination strategy with the aim to contain the spread of the epidemic (2, 3). Recombinant viral vectors have been identified as promising strategies for inducing an anti-EBOV immune response due to their ability to induce potent insert-specific cellular immunity and high levels of antibodies (4). However, vector-based vaccines present major limitations, including the induction and/or pre-existence of anti-vector neutralizing antibodies, which may hamper vaccine’s clinical use (5). Moreover, the restricted use of replicating vaccine platforms like rVSV in target populations where immunosuppressive conditions or co-morbidities (e.g., HIV, TB, and malaria) are common and the need for a strict cold chain storage and transport at −80°C to ensure vaccine stability and biological activity make these vaccines less suitable for large-scale vaccination in sub-Saharan countries.

All current vaccine candidates target the surface EBOV GP, which is the only protein expressed on the virus surface and plays a critical role in EBOV infection (6). EBOV GP is a type I transmembrane protein of 676 amino acids. It is post-translationally cleaved by furin into two subunits (GP1 and GP2) linked by a disulphide bond, and is inserted into the viral membrane. While GP1 mediates attachment to host cells, GP2 is the responsible for fusion of viral and host cell membranes. In the EBOV surface, GP assembles into a trimer of GP1-2 heterodimers that are highly glycosylated and adopt a chalice-like shape. The glycosylation occurs in the mucin-like domains of each monomer, and forms a shield protecting the virus from antibody recognition (6). Humoral responses of EVD epidemic survivors mainly target the GP protein, and anti-GP neutralizing antibodies have been associated with protection against EBOV infection (7–11). Notably, neutralizing mAbs have shown stronger binding to the GP protein in the absence of the mucin-like domain (12, 13) suggesting that its removal could reveal critical epitopes.

A panel of human neutralizing antibodies directed against EBOV GP has been isolated from donors that recovered from EVD, including KZ52 (14), mAb100, and mAb114 (12, 15). The majority of these mAbs show neutralizing activity when administered in combination (16), highlighting the importance of targeting multiple GP epitopes for complete efficacy (17). Despite their proved efficacy, mAb-based treatments have raised concerns in terms of susceptibility to re-infection of the treated survivors (10). The ideal alternative to mAb therapies is represented by the possibility of eliciting neutralizing antibodies *in vivo* through immunization with a highly characterized protein adopting a native-like conformation.

Based on these observations, we propose a native-like recombinant GP protein complex as a promising antigen in order to develop a prophylactic vaccine able to elicit protective/neutralizing antibodies against EBOV infection.

## Materials and Methods

### T-Cell Elispot of ChAd3-EBOZ Volunteers

A T-cell Elispot was performed on volunteers of the ChAd3-EBOZ clinical trial (18) (ClinicalTrials.gov, number NCT02289027). PBMCs (peripheral blood mononuclear cells) were stimulated with 6 pools of 20–22 15-mers peptides covering the sequence of the GP protein, and the response to each pool was calculated independently or as the sum of the responses to the pool of peptides covering the whole GP sequence (pool 1 to 6) with or without subtracting the response due the pool covering the sequence of the mucin-like domain (pool 4). ChAd3-EBOZ clinical trial was reviewed and approved by the local ethics review board (Commission cantonale d**’**éthique de la recherche sur l**’**être humain), by the WHO Research Ethics Review Committee, and by the Swiss regulatory authorities (Swissmedic). All participants provided written informed consent.

### Ebola GP Protein Production and Purification

Chinese Hamster Ovary cells “CHOExpress™” (ExcellGene SA, Monthey, Switzerland), adapted to suspension culture and growing in animal component free media were subcultivated in ProCHO5 medium (Lonza) every 3 or 4 days under orbital shaking at 180 rpm in 50-ml OrbShake tubes (TubeSpin bioreactor 50, TPP, Trasadingen) in an incubator shaker (Kuhner Shaker) set to 37°C and 5% CO_2_. Cells grow to densities of 4 – 8 x 10^6^ cells ml^−1^ under these conditions.

Transfections with cells from expanded seed train cultures of CHOExpress™ cells were executed using ExcellGene’s proprietary gene expression and selection system to obtain stable recombinant CHO cell lines. Stable, recombinant CHO cells were established using puromycin as a selective agent. Productions were done under high density cultures, using a proprietary, antibiotic free, chemically defined, protein-free medium, FlexiCHO^®^ (ExcellGene SA), and proprietary bioreactor-based processing steps under Fed-batch cultures were used to maximize yields. Supernatants were harvested after 10-14 days. For the majority of constructs, expression levels of 10s to 100s of mg l^−1^ were obtained.

Specifically, four EBOV GP constructs were produced in CHO cells (detailed diagrams are reported in **Figure S1**). All had the DNA for the transmembrane and intracellular tail sequence of the protein deleted (“ΔTM”), facilitating the secretion of the products into the culture supernatant of the cell culture. In addition, the “mucin” region of the protein (“ΔMUC”) was deleted in three out of four constructs. The furin-cleavage site was left untouched, since it was assumed that the intracellular cleavage at this position is an essential step in the appropriate folding of the GP protein complex. The “T4” and “GCN4” labelled constructs contain short stretches of additional DNA sequences which were expected to facilitate the assembly of monomeric GP units into trimeric structures. If trimerized as soluble molecule complexes, such a structure would more closely mimic the structure of GP as they are presented on the surface of the EBOV. The T4 and GCN4 sequences are naturally derived protein sequences (19, 20). DNA constructs used for transfection contained “His-tag” encoding DNA, which was inserted to facilitate the downstream purification strategy. Purification was done after clarification and cell removal by buffer exchange and chromatography using a GE Healthcare HiScreen™ NiFF column for His-tagged proteins, following the manufacturer’s recommendation for use.

### Molecular Mass Determination

Multi-angle light scattering was used to assess proteins monodispersity and molecular weight. Between 50 and 100 µg of each protein were separated on a Superose™ 6 increase 10/300 GL column (GE Healthcare) using a HPLC system (Ultimate 3000, Thermo Scientific) coupled in-line to a multi-angle light scattering device (miniDAWN TREOS, Wyatt). Static light-scattering signal was recorded from three different scattering angles. Dn/dc values for the various proteins were determined theoretically according to the molecular mass and the amount of O- and N- glycosylation sites in each protein sequence. The scatter data were analyzed by ASTRA software (version 6.1, Wyatt).

### Circular Dichroism Studies

Circular dichroism (CD) spectra of recombinant constructs were recorded on a JASCO J-815 spectrometer (JASCO Corporation, Tokyo, Japan) equipped with a temperature controller and a 0.1 cm path length cuvette. The measurements were performed in low saline buffer at pH 7.3 and 22°C and at proteins concentration of 250 µg ml^−1^.

For thermal stability profiles, spectra of proteins were registered from 20°C to 90°C, at 10°C intervals. The data were normalized to protein concentrations and expressed in units of molar residue ellipticity. Data analysis and display were performed using GraphPad Prism 7 software. Secondary structure percentages were quantified from the CD data (range 200–250 nm) using BestSel (21, 22).

### Immunological Profiling by ELISA

Recombinant GPs were detected with: the human anti-GP mAb KZ52 (code 0260-001, IBT BioServices); four murine mAbs produced by CEA (EZP01S, EZP08S, EZP16S, EZP35S, all tested for the recognition of MLV-ZEBOV GP Δmuc pseudotypes in the *in vitro* neutralization assays developed in house). The four murine mAbs were demonstrated to recognize four different non-overlapping epitopes, even though each specific epitope is unknown. The four epitopes resulted also to not compete with the epitope recognized by KZ52 (data not shown).

96-well Nunc maxi-sorp plates (code 442404, Thermo Fisher Scientific) were coated with rabbit mAb KZ52 (code Ab 00690-23.0, Absolute Antibody) by incubating overnight at 4°C with 50 µl/well of antibody at 2 µg ml^−1^ in 10 mM of phosphate buffer pH 7.4 (PBS 1×, CHUV). After removal of the coating solution, the coated plates were blocked with 300 µl/well of PBS containing 3% milk powder. Recombinant GP proteins were diluted at 4 µg ml^−1^ in PBS containing 1.5% milk powder and 0.05% Tween 20 (experimental buffer), and samples were dispensed onto the coated wells (50 µl/well). Detection antibodies appropriately diluted at 0.8 µg ml^−1^ in experimental buffer were added to the plate (50 µl/well). Horseradish peroxidase (HRP)–conjugated goat anti-human (code 62-8420, Invitrogen) or anti-mouse (code A0412, Sigma) IgG were then used, according to detection antibodies specificity, as second labeled antibody (diluted 1:1000 in experimental buffer, 50 µl/well). Each incubation step was performed for 1 h at room temperature (RT). After the last incubation step, TMB Substrate Reagent Set (code 555214, BD Biosciences) was added to the plates (50 µl/well) and the color reaction was blocked after 7 min incubation at RT by addition of 0.2 M sulfuric acid (50 µl/well). Absorbance values at 450 and 630 nm were determined on a Tecan Infinite^®^ 200 PRO microplate reader. After each incubation step, plates were washed three times with PBS containing 0.05% Tween 20 (wash buffer) using an automated wash station (Tecan HydroSpeed™) to remove unbound antigen and/or antibody.

### *In Vitro* Neutralization Assay

*In vitro* neutralization assays are based on the use of murine leukemia virus (MLV)-derived retroviral pseudotype expressing envelope proteins of desired viruses. To produce MLV-EBOV pseudotypes, three plasmids were co transfected transiently in Human Embryonic Kidney (HEK) 293T cells using polyethylenimine. Plasmids comprised one encoding Gag Pol proteins from MLV; another the green fluorescent protein (GFP) with a Ψ sequence as an encapsidation signal; and the last one encoding the glycoprotein precursor (GP) of Zaire EBOV (ZEBOV) species. ZEBOV GP1 sequence was deleted of its mucin domain (ΔMUC). Transfected cell supernatants were harvested and clarified 48 h post-transfection, and MLV-ZEBOV GP ΔMUC pseudotypes were concentrated using centrifugation on a sucrose cushion. They were further purified through an ultracentrifugation (Optima XPN80, Beckman) on a continuous sucrose gradient. Purified pseudotypes were then titrated (transducing units, TU ml^−1^) onto VeroE6 cells. The GFP positive cells (i.e., transduced cells) were quantified using FACS analysis (FACSCalibur™, Becton Dikinson).

Mouse monoclonal antibodies (mAbs) were developed by CEA using mice immunized with MLV-ZEBOV GP ΔMUC pseudotypes by lymphocyte fusion with myeloma cells and cloning, according to Kohler and Milstein (23). Their specificity and neutralizing activity were assessed on native EBOV in a BSL-4 laboratory (data not shown).

*In vitro* assays were routinely performed in 96-well plates using MLV-ZEBOV GP ΔMUC pseudotypes and mAbs to evaluate neutralizing potential of mAbs or serum samples. Succinctly, a standard quantity of MLV-ZEBOV GP ΔMUC pseudotypes was incubated with a standard quantity of mAbs under agitation. Then, the mixture was deposited on VeroE6 cells. 48 h post-transduction, cells were fixed with paraformaldehyde and analyzed by flow cytometry to assess GFP fluorescence. To study interactions between recombinant GP and mAbs, neutralizing mAbs (EZP01S, EZP16S, and EZP35S) were pre-incubated at two different concentrations (1 and 10 µg ml^−1^) with various concentrations of GP ΔTM, GP ΔTM-ΔMUC, GP ΔTM-ΔMUC-T4 or GP ΔTM-ΔMUC-GCN4 (10 to 150 µg ml^−1^). The resulting mAb/GP solutions were incubated with pseudotypes, before to be added on VeroE6 cells to evaluate the resulting transduction rate by FACS analysis.

### Direct ELISA With Human Sera

96-well Nunc MaxiSorp plates (code 442404, Thermo Fisher Scientific) were coated overnight at 4°C with 50 µl/well of GP ΔTM, GP ΔTM-ΔMUC, GP ΔTM-ΔMUC-T4, or GP ΔTM-ΔMUC-GCN4 diluted at 0.6 µg ml^−1^ in 10 mM of phosphate buffer pH 7.4 (PBS 1×, CHUV). After removal of the coating solution, the coated plates were blocked with 150 µl/well of PBS containing 3% milk powder. Recombinant GPs were detected with a panel of 10 sera of volunteers from the ChAd3-ZEBOV clinical trial (18), selected amongst the highest responders 28 days after vaccination, and a panel of 10 anonymized EVD survivors. Samples were serially diluted in PBS containing 1.5% milk powder and 0.05% Tween 20 (experimental buffer), and dispensed onto the coated wells (50 µl/well). HRP-conjugated goat anti-human (code 62-8420, Invitrogen) IgG were then used as second labeled antibody (diluted 1:1000 in experimental buffer, 50 µl/well). Each incubation step was performed for 1 h at RT. After the last incubation step, TMB Substrate Reagent Set (code 555214, BD Biosciences) was added to the plates for development (50 µl/well) and the color reaction was blocked after 7 min incubation at RT by addition of 0.2 M sulfuric acid (50 µl/well). Absorbance values at 450 and 630 nm were determined on a Tecan Infinite^®^ 200 PRO microplate reader. After each incubation step, plates were washed three times with PBS containing 0.05% Tween 20 (wash buffer) using an automated wash station (Tecan HydroSpeedTM) to remove unbound antigen and/or antibody. Results were expressed as endpoint titers.

### Competition ELISA

Similarly to what described above, the competition ELISA was performed on a panel of 10 sera derived from the ChAd3-EBOZ clinical trial volunteers and on the panel of 10 EVD survivors from Guéckédou (Guinea). Survivor samples were obtained after informed consent and under approvals from the National Committee of Ethics for Health Research in Guinea (33/CNERS/15) and the Medical Ethics Commission of the State of Hamburg (PV5309). Sera samples, at the dilution giving 50% of the maximal binding to coated T4 or GCN4 trimers, were incubated 1 h at RT with 1:10 serial dilutions of competitor soluble GP ΔTM-ΔMUC monomer or the T4/GCN4 trimers starting with concentrations of 60 µg ml^−1^ or without protein as control (max). The resulting inhibited samples were then dispensed onto the coated wells (50 µl/well), and assay was carried on as previously described. Percentage of inhibition was calculated as: 100-(OD – OD_min_)/(OD_max_ – OD_min_) × 100, where OD_min_ is the signal in well without serum and OD_max_ is the signal resulting from samples incubated without competitor protein. The concentration of soluble inhibitor required to obtain 50% inhibition of IgG binding to the coated protein (EC50) was calculated using the linear function: % inhibition = f [log (protein concentration)].

### Statistical Analysis

Statistical methods are described in figure legends. Unless otherwise stated, analysis was performed using GraphPad Prism 7 (GraphPad Software, La Jolla, CA).

## Results

### T-Cell Elispot of ChAd3-EBOZ Volunteers

**Figure 1:**
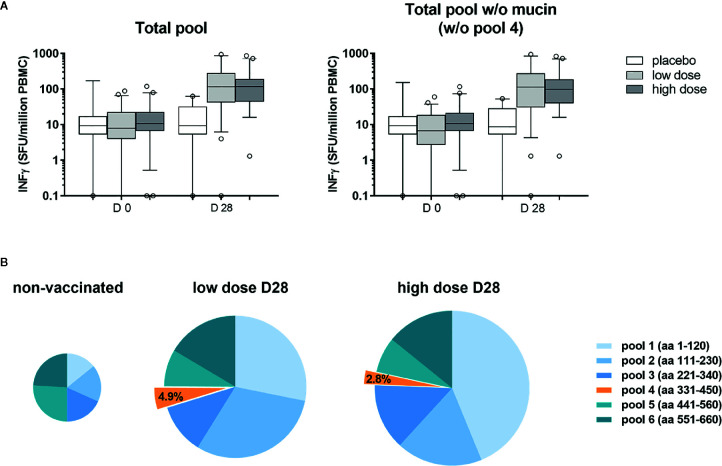
The mucin-like domain was poorly immunogenic for ChAd3-EBOZ volunteers. T-cell Elispot performed on sera from volunteers of the Lausanne clinical trial, whose T-cells were stimulated with a pool of 15-mers overlapping peptides covering the entire sequence of the GP protein [**(A)** left graph] or the same pool without the region corresponding to the mucin-like domain [**(A)**, right graph]. Analysis was performed before vaccination (D0) or 28 days after vaccination (D28) in the placebo group, as well as in the groups of volunteers immunized with the ChAd3-EBOZ vaccine at low dose or high dose. In **(B)** the contribution of each pool to T-cells stimulation is highlighted. Information about the GP region covered by each pool are reported in the figure. The size of the pies correlates with intensity.

PBMC from volunteers of the ChAd3-EBOZ clinical trial (18) were stimulated with six pools of 15-mers overlapping peptides covering the whole sequence of the GP protein (from pool 1 to 6) or the GP protein lacking the mucin-like domain (represented by pool number 4, which was not included for stimulation) (**Figure 1A**) in order to evaluate the contribution of the mucin-like domain to GP immunogenicity. As shown in **Figure 1A**, PBMCs stimulation with the full-length protein or the mucin-deleted one produced comparable results in terms of stimulation, suggesting the mucin-like domain was poorly immunogenic for ChAd3-EBOZ volunteers. In **Figure 1B**, we dissected the individual contribution of each pool of peptides to PBMCs stimulation in non-vaccinated individuals (placebo groups and all the D0 individuals) or in volunteers receiving the vaccine at low or high dose. Considering the minimal contribution of the mucin-like domain to T-cell immunogenicity, we considered the possibility to delete this domain. Moreover, the presence of the heavily glycosylated mucin-like domain appears to play a role in immune evasion by shielding the virus from efficient humoral responses in EVD infected individuals (24), and we considered this as an additional argument to support the production of a deleted-construct.

### Production and Characterization of Soluble EBOV GP Trimers

We designed and produced in Chinese hamster ovary (CHO) cells a recombinant GP lacking the transmembrane domain (GP ΔTM) for facilitated secretion and, as previously discussed, a mutant construct with deletion of the mucin-like domain together with the transmembrane deletion (GP ΔTM-ΔMUC). On the EBOV surface, the GP protein is present as a non-covalently linked, highly glycosylated chalice-like shaped trimer. For this reason, additional variants of GP ΔTM-ΔMUC were engineered by fusing trimerization motifs to their C-terminus to induce and stabilise the formation of GP ΔTM-ΔMUC trimers. Specifically, a trimerization domain from the C-terminus of bacteriophage T4 fibritin (T4) (25, 26) and the sequence of the GCN4 transcription factor (GCN4) (27) were used.

**Figure 2:**
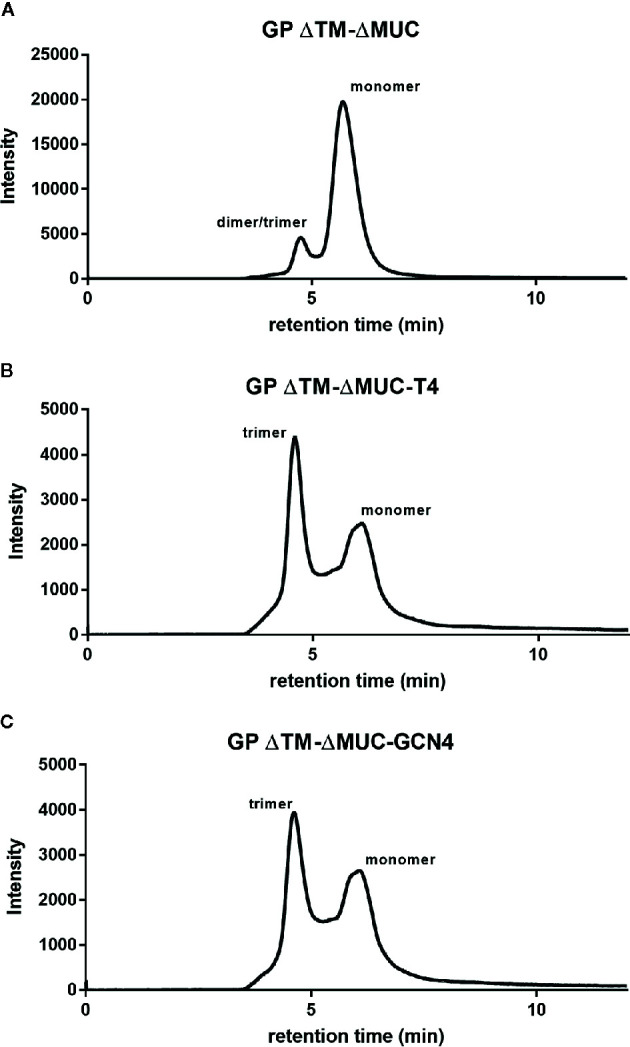
The insertion of a C-terminal trimerization motif drives the trimerization of the GP ΔTM-ΔMUC protein. SEC profiles of **(A)** GP ΔTM-ΔMUC, **(B)** GP ΔTM-ΔMUC-T4, and **(C)** GP ΔTM-ΔMUC-GCN4.

Considering the importance of having a fully characterized protein for data interpretation and future (pre-)clinical studies, we applied a panel of orthogonal techniques to characterize the structural integrity of the recombinant proteins and to check their ability to assume a correct trimeric conformation. As shown in **Figure 2**, the insertion of a trimerization motif favored the trimeric structure in solution (panels B and C), when compared to the profile exhibited by GP ΔTM-ΔMUC (panel A), which is mostly monomeric. The fractions corresponding to the trimeric molecules GP ΔTM-ΔMUC-T4 and GP ΔTM-ΔMUC-GCN4 were isolated and purified. The determination of the absolute molecular weight of the four GP variants provided the analytical evidence that inclusion of the T4 or GCN4 trimerization domains at the C-terminus of the GP sequence drove the trimerization of the GP ΔTM-ΔMUC monomer (peak 2) in CHO cells (**Table 1** and **Figure S2**). GP ΔTM-ΔMUC peak 1 likely represent the formation of a dimer or an incomplete trimer. Notably, the peak of GP ΔTM has double the size of GP ΔTM-ΔMUC monomer (peak 2), which is consistent with the mucin-like domain contributing for almost half the mass of the GP protein (28, 29).

**Table 1:** SEC-MALS absolute determination of the proteins’ molecular weight.

	Peak 1	Peak 2
GP ΔTM	196 kDa	–
GP ΔTM-ΔMUC	240 kDa	97 kDa
GP ΔTM-ΔMUC-T4	316 kDa	–
GP ΔTM-ΔMUC-GCN4	296 kDa	–

**Figure 3:**
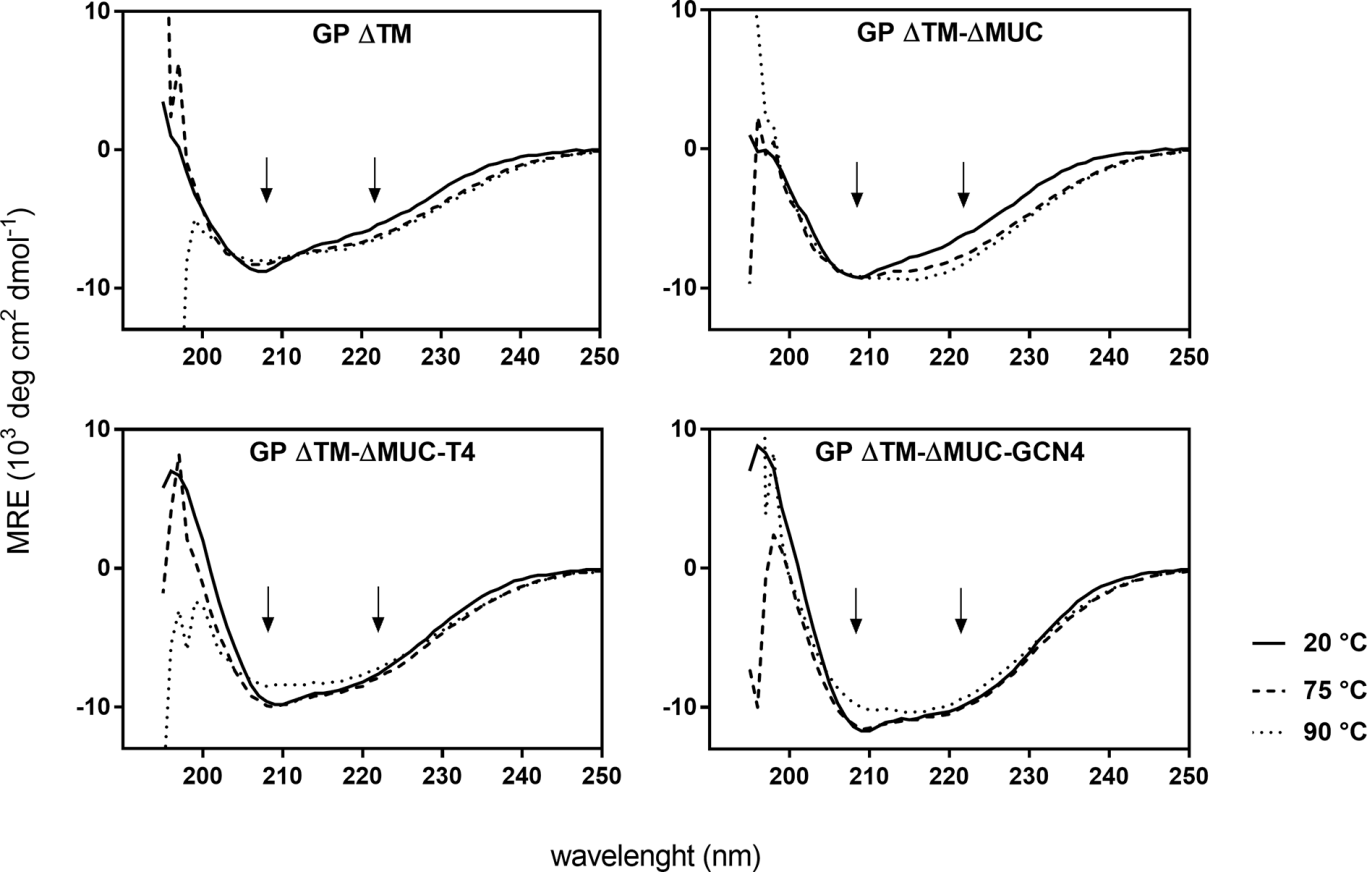
The insertion of a C-terminal trimerization motif favors the formation of the α-helical bundle and enhances the thermal stability of the recombinant GP. Temperature dependent CD spectra of GP ΔTM, GP ΔTM-ΔMUC, GP ΔTM-ΔMUC-T4, and GP ΔTM-ΔMUC-GCN4. Spectra were registered between 190 and 250 nm and between 20°C and 90°C at 5°C interval. For clarity, only spectra at 20°C, 75°C, and 90°C are shown. The arrows indicate the double minima at 208 and 222 nm, spectral signature typical of an α-helical secondary structure.

The secondary structure profiles of the four GP proteins displayed a spectral signature typical of a α-helical secondary structure, with double minima at 208 and 222 nm, the former being less evident because of the contribution of β-sheets present in the head of the protein (**Figure 3**). The percentage contribution of each secondary structure was also calculated using BestSel algorithm and results are reported in **Table S1**. The CD profiles of the four GP proteins are consistent with the α-helical bundle motifs typical of class I transmembrane envelope viral GPs that is used to drive fusion of viral and host membranes (30). Interestingly, the two trimeric proteins exhibited a stronger contribution of the α-helical secondary structure with respect to the monomers, which is due to the insertion of the trimerization motifs favoring the formation of the α-helical coiled coil. Changes in secondary structures of proteins at different temperatures were also investigated. GP ΔTM-ΔMUC-T4 and GP ΔTM-ΔMUC-GCN4 appeared to be stable up to 75°C and showed the lowest impact in structural changes at the various temperatures tested (**Figure 3**), confirming that the stability of the soluble GP protein is enhanced by the addition of T4 or GCN4 sequences.

### Immunological Profiling of Soluble EBOV GP Antigens

**Figure 4:**
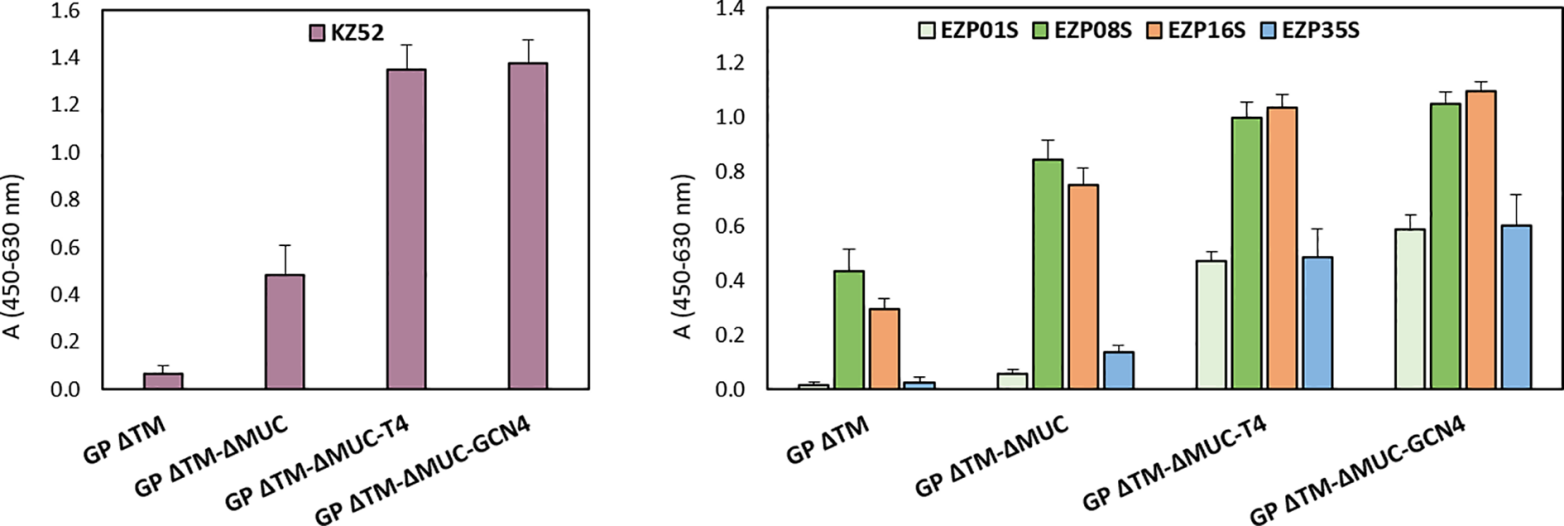
Recombinant GP trimers show an increased breadth of reactivity with a panel of conformational mAbs. Sandwich ELISA analysis of GP ΔTM, GP ΔTM-ΔMUC, GP ΔTM-ΔMUC-T4, and GP ΔTM-ΔMUC-GCN4. The graphs show the results of the sandwich ELISA with human KZ52 (left) or a panel of mouse mAbs (right) as detection reagents. Average blank values were subtracted from sample values. Column heights represent the mean values of 3 independent assays, with corresponding standard deviations.

The screening and immunological profiling of the designed immunogens were carried out by ELISA in order to assess the recognition of critical neutralizing epitopes. Our aim was to confirm the structural integrity of the produced proteins and their ability to be recognized by monoclonal antibodies recognizing conformational epitopes including KZ52 (14), and a panel of murine neutralizing mAbs produced in-house. In order to minimize the impact on protein structures introduced by adsorption to the ELISA plate, the immunological profiling of the recombinant proteins was performed in a sandwich ELISA configuration, using a chimeric version of mAb KZ52 produced in rabbit as capture antibody. Surprisingly, signal was detected with the antibody pair rabbit KZ52/human KZ52 for all the tested mucin-deleted constructs (**Figure 4**). Since the epitope recognized by KZ52 is known to be located in the GP protomer (6), this was identified as an indirect proof of the ability of the GP ΔTM-ΔMUC to rearrange in a dimeric, if not trimeric, conformation when in solution (as noticed also in **Figure 2** and **Table 1**). The reactivity of trimers in this assay was significantly higher than that of the two monomeric molecules. Considering that KZ52 epitope is located in the GP promoter and bridges both GP1 and GP2 subunits, we could hypothesize that our monomeric recombinant GPs do not express this epitope in its right conformation and that the epitope might be covered by the mucin-like domain in GP ΔTM.

We extended the immunological profiling of the antigens using a panel of four conformational mouse mAbs produced in house and already tested for the recognition of pseudotypes expressing ZEBOV GP ΔMUC in an *in vitro* neutralization assay. Among these mAbs, listed in the graph of **Figure 4**, EZP01S, EZP16S, and EZP35S were known for their *in vitro* neutralizing activity, with PRNT50 of 6.9, 2.7, and 2.4 µg/ml^−1^, respectively. Overall, the mucin-deleted versions of the protein were recognized with higher efficiency by all the tested mAbs, confirming the initial hypothesis of the mucin-like domain to act in limiting access of mAbs to their corresponding epitopes. Remarkably, the immunological profiling of the engineered variants showed an increased breadth of reactivity with this panel of mAbs. This increase is particularly evident for mAbs KZ52, EZP01S, and EZP35S. These three antibodies are probably quaternary specific and have more affinity for their respective epitopes when the protein is in the trimeric conformation.

**Figure 5:**
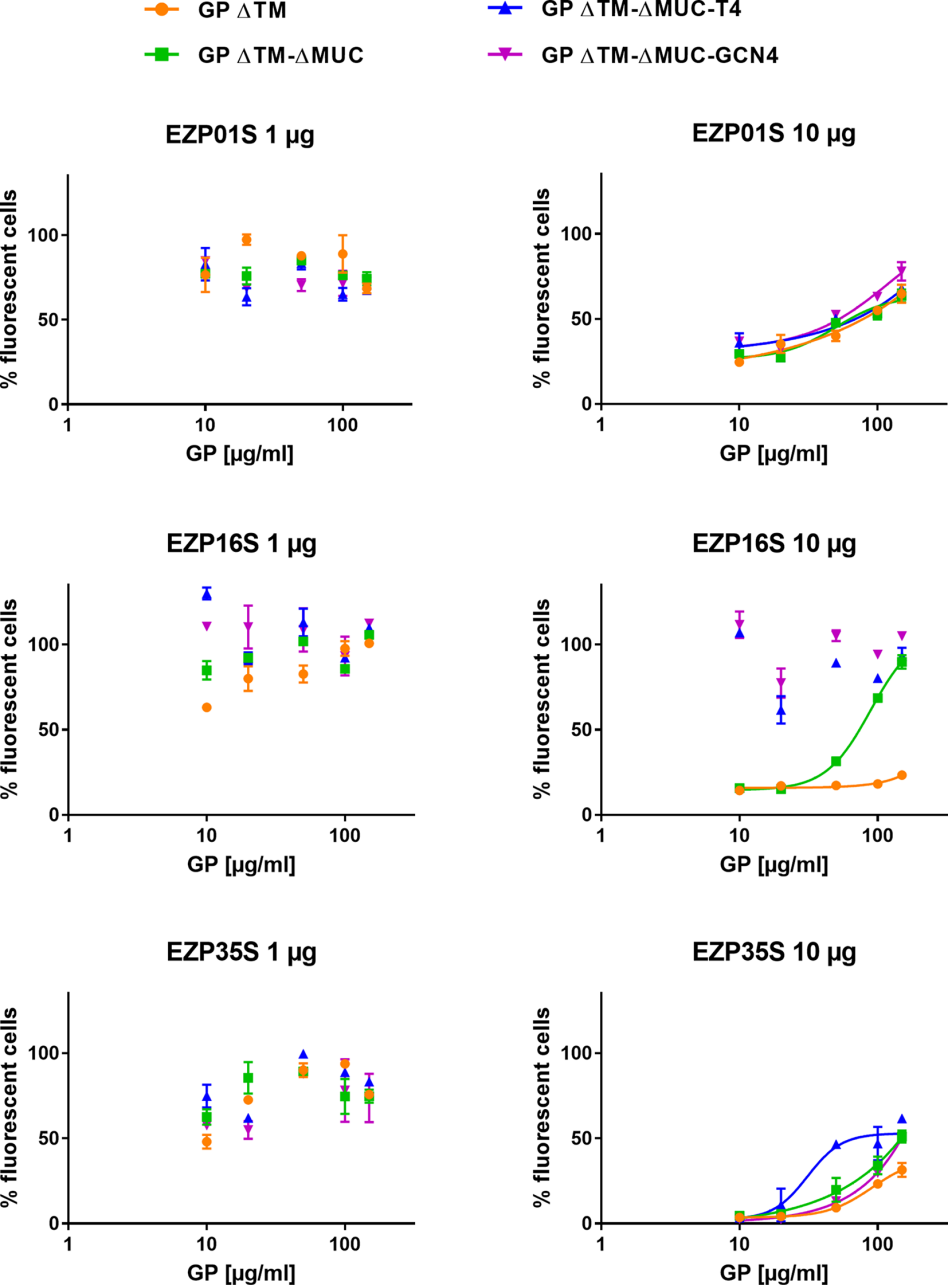
Recombinant GPs are recognized by neutralizing mAbs in solution. An evaluation of the inhibitory activity of GP ΔTM, GP ΔTM-ΔMUC, GP ΔTM-ΔMUC-T4, and GP ΔTM-ΔMUC-GCN4 was performed on a pseudo-type infection assay in presence of EZP01S, EZP16S, or EZP35S as neutralizing antibodies. When possible, dilution points were fitted with a four-parameter dose-response curve.

The effect of the EBOV GP variants in inhibiting the neutralizing activity of mAbs in a transduction assay was assessed on a murine leukemia virus-derived retroviral pseudotype platform. The principle of the test was to evaluate the ability of mAbs to neutralize the transduction of cells made by pseudoviruses expressing the ZEBOV GP ΔMUC. We tested if our recombinant GPs could be recognized by neutralizing mAbs thus preventing/avoiding their inhibition activity on the transduction assay. To answer this question, neutralizing antibodies EZP01S, EZP16S, and EZP35S were tested at two different concentrations (1 and 10 µg ml^−1^), corresponding to an 80% and 0% of transduced cells in the pseudotype assay, against various concentrations of recombinant GPs. As indicated from the left panels in **Figure 5**, mAbs at the lowest concentrations captured the soluble EBOV GPs leading to a 100% cells transduction by pseudotypes. The main differences are evident in the right panels with the highest mAb concentrations. In this case, mAbs EZP01S and EZP35S showed to be equally sensitive to GP monomers and trimers, suggesting that the recognized epitope is similarly accessible in both protein conformations. On the other hand, mAb EZP16S recognizes an epitope that is highly accessible in the trimers, and probably covered by the mucin-like domain given the fact that in presence of the GP ΔTM monomer cell transduction is completely inhibited (**Figure 5**). This experiment presented us with an outcome that is opposite from what shown in **Figure 4** where binding of EZP01S and EZP35S was more likely to happen with trimers rather than with monomeric GP, and EZP16S was similarly sensitive to monomers and trimers. However, such comparison is biased by the different kinetic of the two experiments. In ELISA, the binding is happening over an array of coated antibodies that force the GP in a less natural position. On the contrary, in the transduction assay the GP protein is expressed over the surface of pseudoviruses thus being more exposed and accessible to mAbs similarly to what happens *in vivo*.

### Candidate Selection for Pre-Clinical Studies

**Figure 6:**
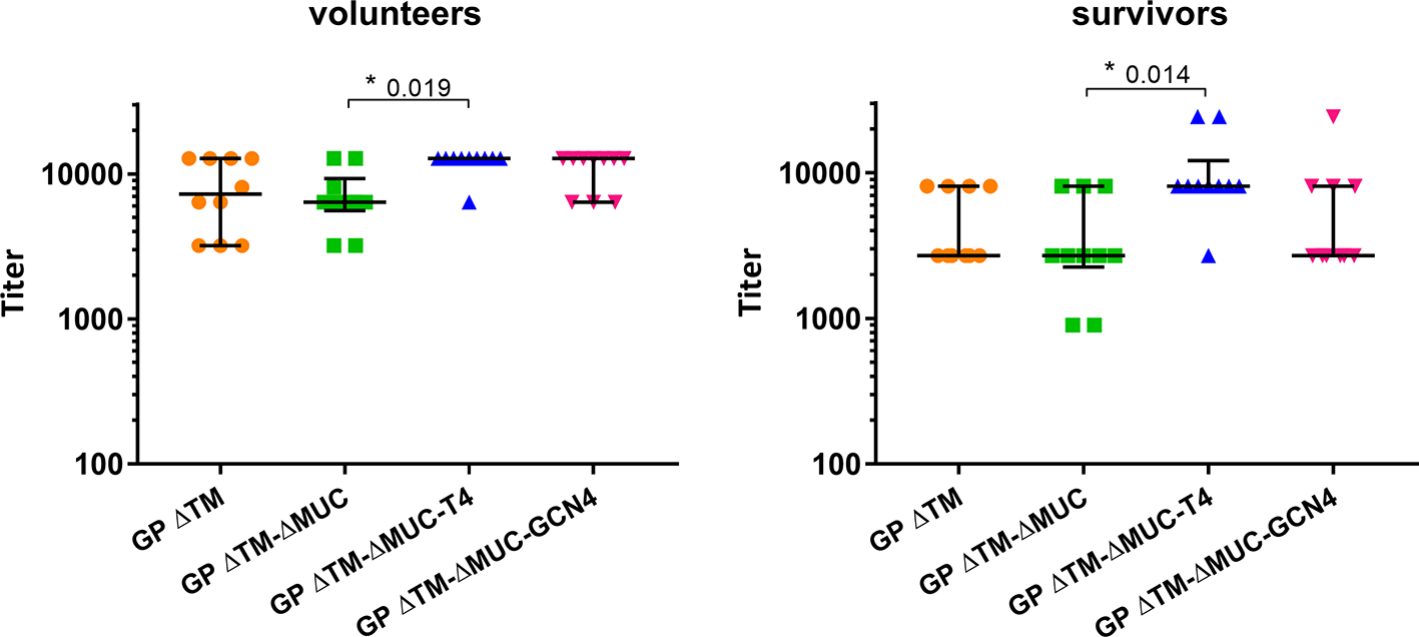
The T4 trimer is mostly recognized by sera of clinical trial volunteers and Ebola disease survivors. A panel of 10 sera derived from clinical trial volunteers (left graph) and a panel of 10 Ebola virus survivors (right graph) were analyzed in direct ELISAs to test the recognition of GP ΔTM, GP ΔTM-ΔMUC, GP ΔTM-ΔMUC-T4, and GP ΔTM-ΔMUC-GCN4. Median values are shown together with interquartile ranges. Differences among groups were analyzed with a Kruskal-Wallis one-way ANOVA for multiple comparisons.

**Figure 7:**
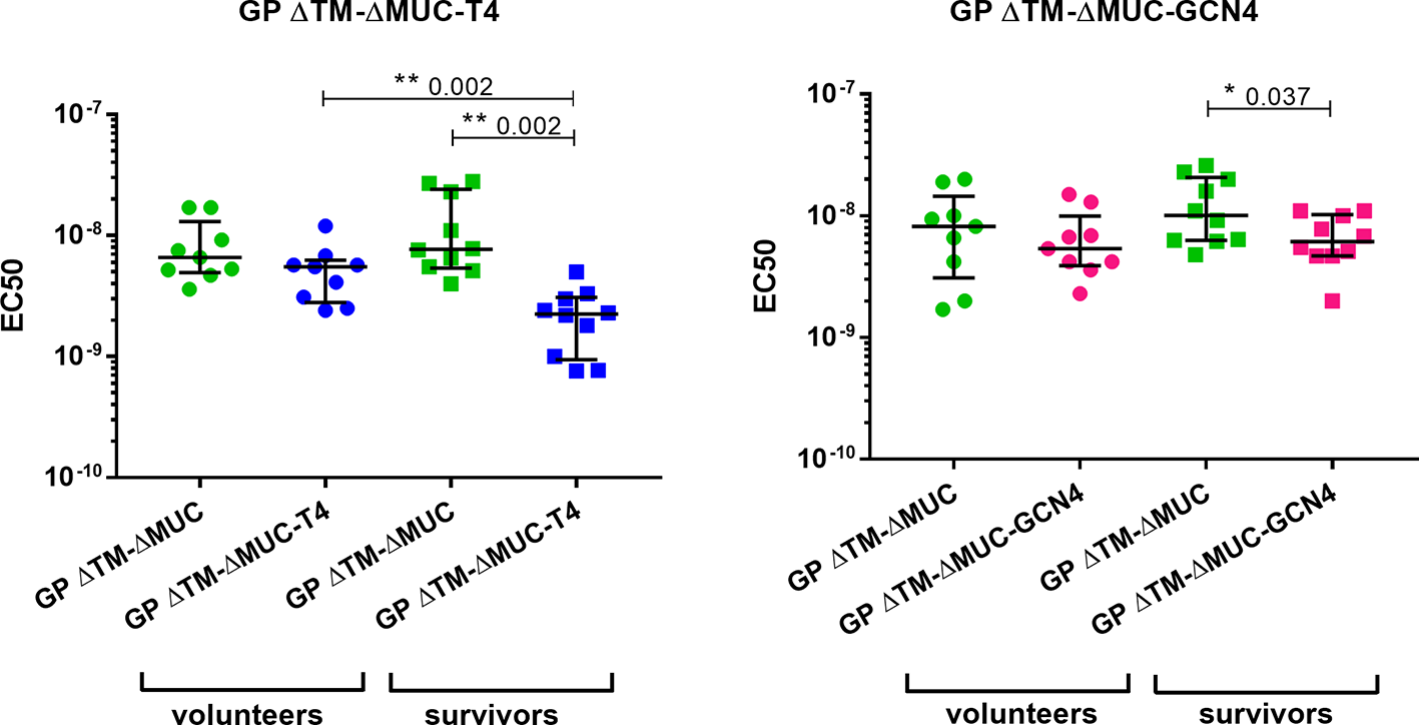
The T4 trimer shows the highest apparent affinity (lowest EC50) for sera of clinical trial volunteers and Ebola disease survivors. A panel of 10 sera obtained from clinical trial volunteers (dots) and a panel of 10 Ebola virus survivors (squares) were analyzed in a competition ELISA to test their respective affinities for the monomer GP ΔTM-ΔMUC (green) or for the GP ΔTM-ΔMUC-T4 (blue) or –GCN4 (pink) trimers. Median values with inter-quartile range values are represented. Statistical significance among groups was evaluated by means of the Wilcoxon test (among the same group of individuals) and the Mann-Whitney test (comparison of volunteers vs. survivors).

Further investigations were directed to the selection of the most promising ΔMUC candidate. A panel of 10 sera from ChAd3-EboZ trial volunteers (18) was tested in a direct ELISA against the recombinant GPs and the antibody responses were compared with responses of a panel of 10 EVD survivors (**Figure 6**). The GP ΔTM-ΔMUC-T4 protein was best recognized by sera from both volunteers and survivors. The two sera panels were also compared in a competition ELISA against various concentrations of the monomer GP ΔTM-ΔMUC or the corresponding T4 and GCN4 trimers (**Figure 7**). Since the panel of survivors showed a higher affinity for T4 trimers, we identified this protein as a most promising antigen candidate for an EBOV GP-based vaccine, even though proper candidate selection should require recombinant GPs to be tested in further pre-clinical studies.

## Discussion

Currently, viral vector-based vaccines lead vaccine research against EVD and the amount of published work focusing on preclinical and clinical studies using recombinant EBOV GP proteins is limited and mainly based on protein production in insect cells (31–33). A recent publication from Rutten and colleagues (34) described the successful insertion of stabilizing mutations for the expression of stabilized GP trimers in human cells. In the present study we instead followed the structure-based approach that led to recent advances in vaccine development for two distinct pathogens, RSV and HIV, which have yielded promising results in terms of induction of neutralizing antibodies when the relevant surface GPs were engineered to present a native-like pre-fusion conformation (35–37). Similar approaches were also applied for the production of EBOV GP trimers to be used mainly for structural biology studies aimed to investigate the formation of complexes between GP and neutralizing antibodies (12, 38) or other therapeutic molecules (26, 39). With these results as a proof-of-concept, we aimed at the production of a stabilized and soluble native-like EBOV GP trimer. Specifically, four variants of soluble ZEBOV GP proteins were engineered and secreted into the supernatant of bioreactors using highly efficient and proprietary technologies for transient and stable expression (40–45). For the first time to our knowledge, CHO cells were exploited for recombinant production of EBOV GP proteins, bringing many advantages such as high yield and the potential for large-scale operation in manufacturing. Moreover, mammalian cell culture systems and CHO cells in particular guarantee post-translational processing and a protein glycosylation machinery highly similar to that in human. We produced GP trimers retaining a stable native-like structure and recognized by virus neutralizing mAbs, even upon removal of the mucin-like domain. In CHO cells, the mucin-deleted constructs appeared to efficiently execute the furin proteolysis step that occurs *in vivo*, thus enhancing the presentation of conserved neutralizing epitopes implicated in receptor binding (33, 46), while removing epitopes located in the glycan cap and mucin-like domain that are mostly linear and non-neutralizing (47). Indeed, the performed experiments confirmed that the recombinant proteins were retaining the conformation of the epitope recognized by several neutralizing mAbs. In particular, the mucin-like domain appeared not to be critical for the retention of the native conformation of the GP protein, and its removal allowed unmasking of neutralizing epitopes. Protein engineering efforts to stabilize the formation of GP trimers were focused on the mucin-deleted version of GP because of this higher reactivity with all the antibodies tested. Globally, the strong biochemical and immunological profiling carried out on vaccine candidates presented here strongly supports the importance of native-like antigen conformation for induction of neutralizing antibodies.

The here produced and characterized native-like proteins will help to overcome problems inherent to virus vector-based vaccines offering additional opportunities to design novel and more efficacious clinical trials with prime-boost regimens for prophylactic vaccines against EBOV. Host pre-existing immunity is indeed the main limiting factor of vector-based vaccines, preventing their use in homologous prime-boost immunization strategies that would likely favor the induction of a stronger and extended protection. Also, considering that lately many viruses are being used as vaccine vectors, the heterologous prime-boost could easily lead to a shortage of available vector vaccine platforms. Multiple exposure to the same antigen would help to increase the durability of protection and the quality of induced antibodies.

Antigens purity and homogeneity are fundamental in terms of safety and efficacy of the final vaccine product. In this perspective, vaccination with a recombinant protein allows the control and characterization of the conformation adopted by the antigen—which is very difficult to determine in the current viral vector-based vaccine candidates—thus representing a desired improvement in terms of safety. Furthermore, a pure and well-defined antigen facilitates the characterization studies of the induced immune response. Recombinant GP vaccines may be easier to produce at higher yields than virus-vector based vaccines, particularly if manufactured in an industrial host system such as CHO cells. Moreover, the remarkable structural and thermal stability demonstrated by our antigens is of fundamental interest in the process of candidate selection for future development of a vaccine intended to be deployed in third world countries.

Nevertheless, we acknowledge that purified recombinant antigens are likely poorly immunogenic and proper adjuvants will be required to balance the removal of natural immune-stimulatory components of the pathogen with the aim to enhance an immune response similar to the one triggered by natural infection.

In conclusion, the results discussed here demonstrate the possibility to obtain soluble EBOV GP trimeric constructs with superior antigenic profiles and the potential to be recognized as valuable antigen candidates for the development of an efficacious prophylactic EBOV vaccine. The possibility to produce a protein vaccine based on a native-like candidate highly recognized by neutralizing monoclonal antibodies and with high affinity for survivors-derived antibodies would confer a great advantage in terms of induction of protective antibodies through vaccination. For this reason, the trimeric soluble recombinant antigens presented here are currently being tested in pre-clinical animal models for immunogenicity and induction of neutralizing antibody responses.

## Data Availability Statement

All datasets presented in this study are included in the article/**Supplementary Material**.

## Ethics Statement

The ChAd3-EBOZ vaccine trial was reviewed and approved by the local ethics review board (Commission cantonale d’éthique de la recherche sur l’être humain), by the WHO Research Ethics Review Committee, and by the Swiss regulatory authorities (Swissmedic). The studies involving human participants from Guinea were reviewed and approved by National Committee of Ethics for Health Research in Guinea (33/CNERS/15) Medical Ethics Commission of the State of Hamburg (PV5309). All patients/participants provided their written informed consent to participate in the studies.

## Author Contributions

Conceived the study: VA, DK, MW, FW, BC, and FS. Performed the experiments: VA, DK, CF, FG, and SW. Analyzed the data: VA, DK, CF, FG, SW, and FS. Contributed with reagents/material: CM-F. Writing—original draft: VA. Writing—review and editing: VA, FW, CF, FG, SW, CM-F, LB, GC, and FS. All authors contributed to the article and approved the submitted version.

## Funding

The present work has been funded within the IMI-funded PEVIA project (project ID 116088) with funding for the Swiss Partners obtained from the Secretary of State for Education, Research and Innovation. This work was also partially funded by a grant of the German Research Foundation (DFG) to CM-F (MU 3565/3-0). The funders had no role in study design, data collection and interpretation, or the decision to submit the work for publication.

## Conflict of Interest

VA, DK, FW, BC, and FS are co-authors of an international patent. EBOLA Vaccine Compositions and Methods of Using Same, Inventors: Kiseljak, et al., publication no. WO 2020/044267, March 5, 2020. DK, MJW, FMW were employed by the company ExcellGene.

The remaining authors declare that the research was conducted in the absence of any commercial or financial relationships that could be construed as a potential conflict of interest.
