# Supplementary material for: Designs and Characterization of Subunit Ebola GP Vaccine Candidates: Implications for Immunogenicity
Source: Front Immunol. 2020 Nov 4;11:586595. doi: 10.3389/fimmu.2020.586595 (PMC7672190; doi:10.3389/fimmu.2020.586595)
Supplement: Supplementary file 1 [file DataSheet_1.docx]

Supplementary Material

**Figure S1. Diagram of the Ebola GP proteins designed for production in CHO cells.** Each optimised expression cassettes contain the DNA sequence derived from a human IgG1 encoding for the Leader Peptide, a CHO codon optimised GP under deletion of the transmembrane and intracellular sequence. The gap in the diagrams indicate a site where, within cells furin, a cellular protease, will cleave the protein into two separate peptide segments. The S-S labelled lines indicate positions where, through intramolecular disulphide bridges the individual peptides are linked and keep the two sections of the GP protein as a monomer together. Diagrams show:

A) GP ΔTM;

B) GP ΔTM-ΔMUC with deletion of the sequence encoding the mucin region;

C) GP ΔTM-ΔMUC-T4 with deletion of the sequence encoding the mucin region and addition of a T4 trimerization peptide at the C-terminus having as sequence *GSGYIPEAPRDGQAYVRKDGEWVLLSTFLGT*;

D) GP ΔTM-ΔMUC-GCN4 with deletion of the sequence encoding the mucin region and addition of a GCN4 trimerization peptide at the C-terminus having as sequence *SGKQIEDKIEEILSKIYHIENEIARIKKLIG.*


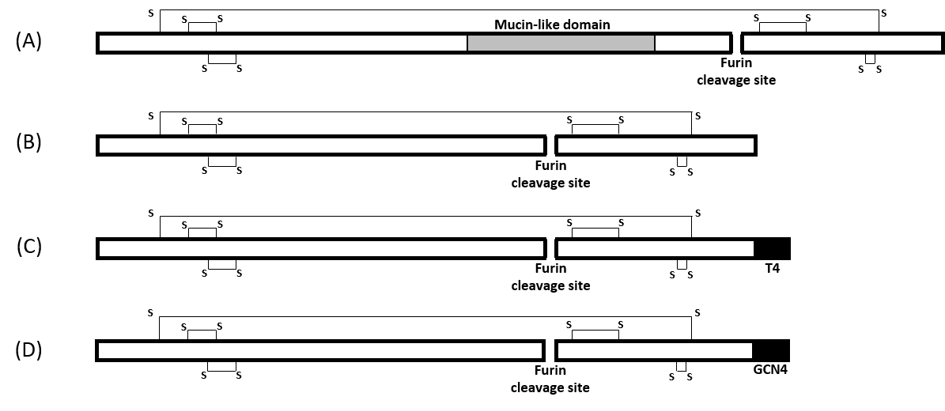


**Figure S2.** **SEC-MALS absolute determination of the proteins’ molecular weight.** Size exclusion chromatography with multi-angle laser light scattering (SEC-MALS) analysis of EBOV GP proteins. The refractive index (light scattering signal, LS) of each protein is plotted as a function of time (black line). Over the peak region, the molecular weight (MW) of the protein (red or blue lines) is calculated from the light scattering measurements.


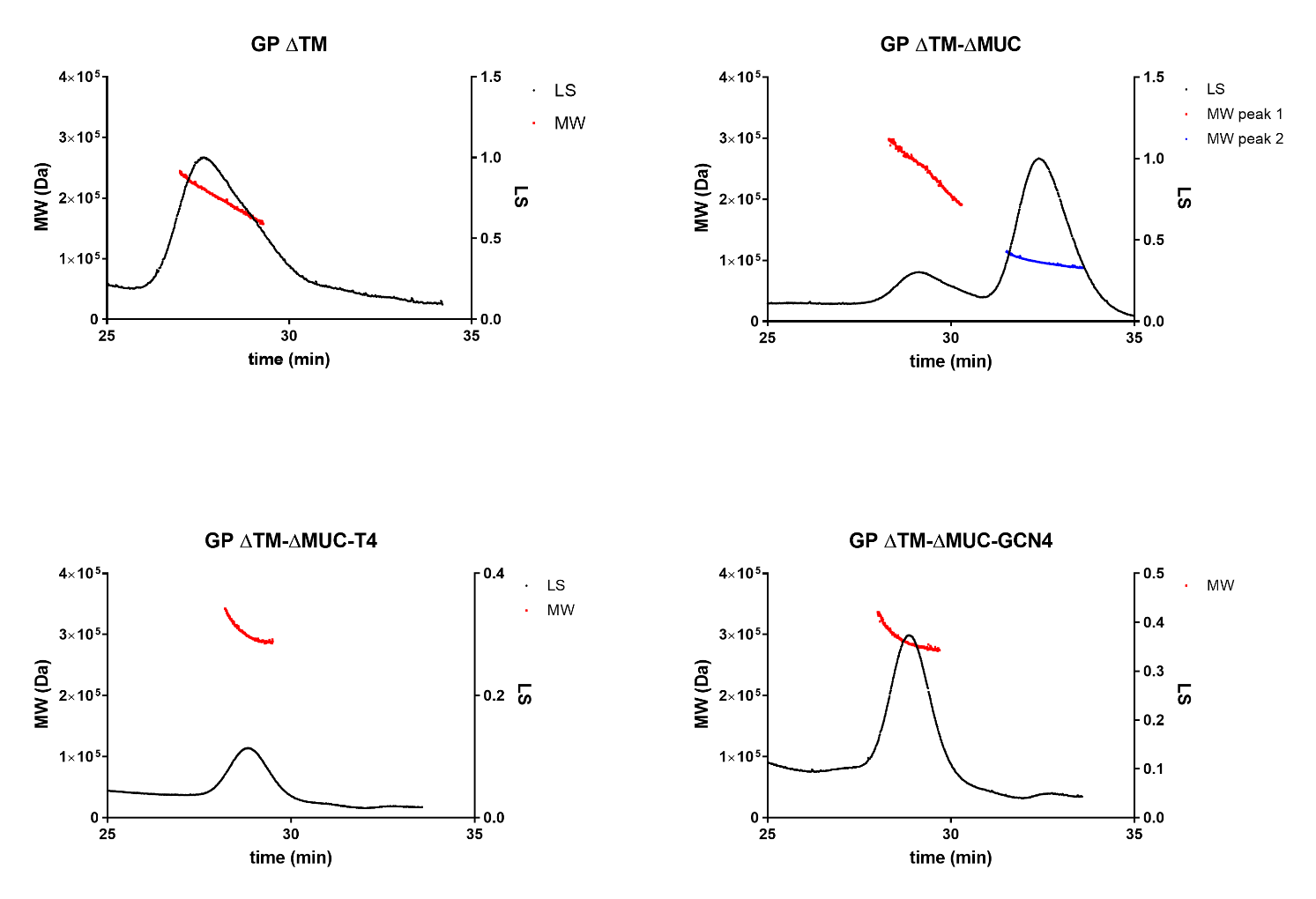


**Table S1:** Secondary structure percentages quantified for recombinant proteins from the CD data using BestSel.

|  | GP ΔTM | GP ΔTM-ΔMUC | GP ΔTM-ΔMUC-T4 | GP ΔTM-ΔMUC-GCN4 |
| --- | --- | --- | --- | --- |
| Helix | 16.8 | 18.4 | 21.5 | 25.8 |
| Beta-sheet | 27.1 | 26.9 | 27.7 | 24.9 |
| Turn | 13.2 | 14.2 | 11.0 | 13.1 |
| Other (coil) | 42.8 | 40.5 | 39.8 | 36.2 |
| Total | 99.9 | 100 | 100 | 100 |
